# Anti-proliferative Effects of Nucleotides on Gastric Cancer via a Novel P2Y6/SOCE/Ca^2+^/β-catenin Pathway

**DOI:** 10.1038/s41598-017-02562-x

**Published:** 2017-05-26

**Authors:** Hanxing Wan, Rui Xie, Jiangyu Xu, Jialin He, Bo Tang, Qingqing Liu, Sumin Wang, Yanjun Guo, Xin Yang, Tobias Xiao Dong, John M. Carethers, Shiming Yang, Hui Dong

**Affiliations:** 1 Department of Gastroenterology, Xinqiao Hospital, Third Military Medical University, Chongqing, China; 2 Department of Gastroenterology, Affiliated Hospital to Zunyi Medical College, Zunyi, China; 3 Department of Medicine, University of California, San Diego, California USA; 4 Department of Internal Medicine, University of Michigan, Ann Arbor, Michigan USA

## Abstract

Although purinegic signaling is important in regulating gastric physiological functions, it is currently unknown for its role in gastric cancer (GC). We demonstrate for the first time that the expression of P2Y6 receptors was markedly down-regulated in human GC cells and primary GC tissues compared to normal tissues, while the expression of P2Y2 and P2Y4 receptors was up-regulated in GC cells. Moreover, the expression levels of P2Y6 receptors in GC tissues were correlated to tumor size, differentiation, metastasis to lymph nodes, and the survival rate of the patients with GC. Ncleotides activated P2Y6 receptors to raise cytosolic Ca^2+^ concentrations in GC cells through store-operated calcium entry (SOCE), and then mediated Ca^2+^-dependent inhibition of β-catenin and proliferation, eventually leading to GC suppression. Furthermore, UTP particularly blocked the G1/S transition of GC cells but did not induce apoptosis. Collectively, we conclude that nucleotides activate P2Y6 receptors to suppress GC growth through a novel SOCE/Ca^2+^/β-catenin-mediated anti-proliferation of GC cells, which is different from the canonical SOCE/Ca^2+^-induced apoptosis in other tumors.

## Introduction

Extracellular nucleotides (such as ATP and UTP) are not only energy sources but also ubiquitous messengers that regulate cell growth, differentiation, cytokine release and cytotoxicity^1–4^. They generate biological responses by acting on purinergic receptors to produce a series of intracellular signaling pathways^5–7^. In purinergic receptor family, there are P1 and P2 receptors. The latter are further divided into the ligand-gated ion channel P2X receptors and G-protein-coupled receptors (GPCRs) P2Y, which are subdivided into eight subtypes (P2Y1, 2, 4, 6 and P2Y11–14). P2Y6 receptors, one member of P2Y receptors highly activated by UTP and UDP, mediate various human physiological and pathological processes^8–13^. Over recent years, nucleotides and purinergic receptors have been the focus of increasing attention by immunologists and oncologists since ATP and UTP are found to modulate tumor growth in various tumor models^14, 15^. However, their roles in the tumorigenesis of upper gastrointestinal tract are largely unknown.

P2Y receptors mainly activate phospholipase C (PLC) through G_q/11_ proteins to generate inositoltrisphosphate (IP_3_). The latter can trigger Ca^2+^ release from intracellular stores (sarcoplasmic/endoplasmic reticulum, S/ER), in turn inducing extracellular Ca^2+^ entry^16^. This mechanism is referred to as the store-operated Ca^2+^ entry (SOCE)^17, 18^. It has been widely accepted that STIM1 and Orai1 are two key molecular components of SOCE to play a prominent role in controlling cytosolic Ca^2+^ ([Ca^2+^]_cyt_) homeostasis in normal cells^19^. However, abnormal SOCE is found to be implicated in several human diseases, such as tumorigenesis^20–23^. Growing lines of evidence indicate that an aberrant P2Y receptor-mediated Ca^2+^ signaling may be involved in growth inhibition and apoptosis of some cancer cells^24^. Unfortunately, their roles and corresponding Ca^2+^ signaling in gastric cancer (GC) have not been explored so far.

GC is the second leading cause of cancer-related death worldwide. Currently, GC is hard to prevent and/or cure due to our poor understanding of its pathogenesis and difficulty in the early diagnosis. Therefore, in the present study we investigated the expression of P2Y receptors, specifically P2Y6 receptor subtype in human GC, their roles in GC development and the underlying molecular mechanisms. We demonstrate for the first time that UTP and UDP suppress GC growth via P2Y6 receptors-mediated SOCE/Ca^2+^ signaling to inhibit β-catenin, which might be a potential strategy in the prevention/treatment of GC.

## Results

### P2Y6 expression is reduced in human primary GC tissues and cells

**Figure 1:**
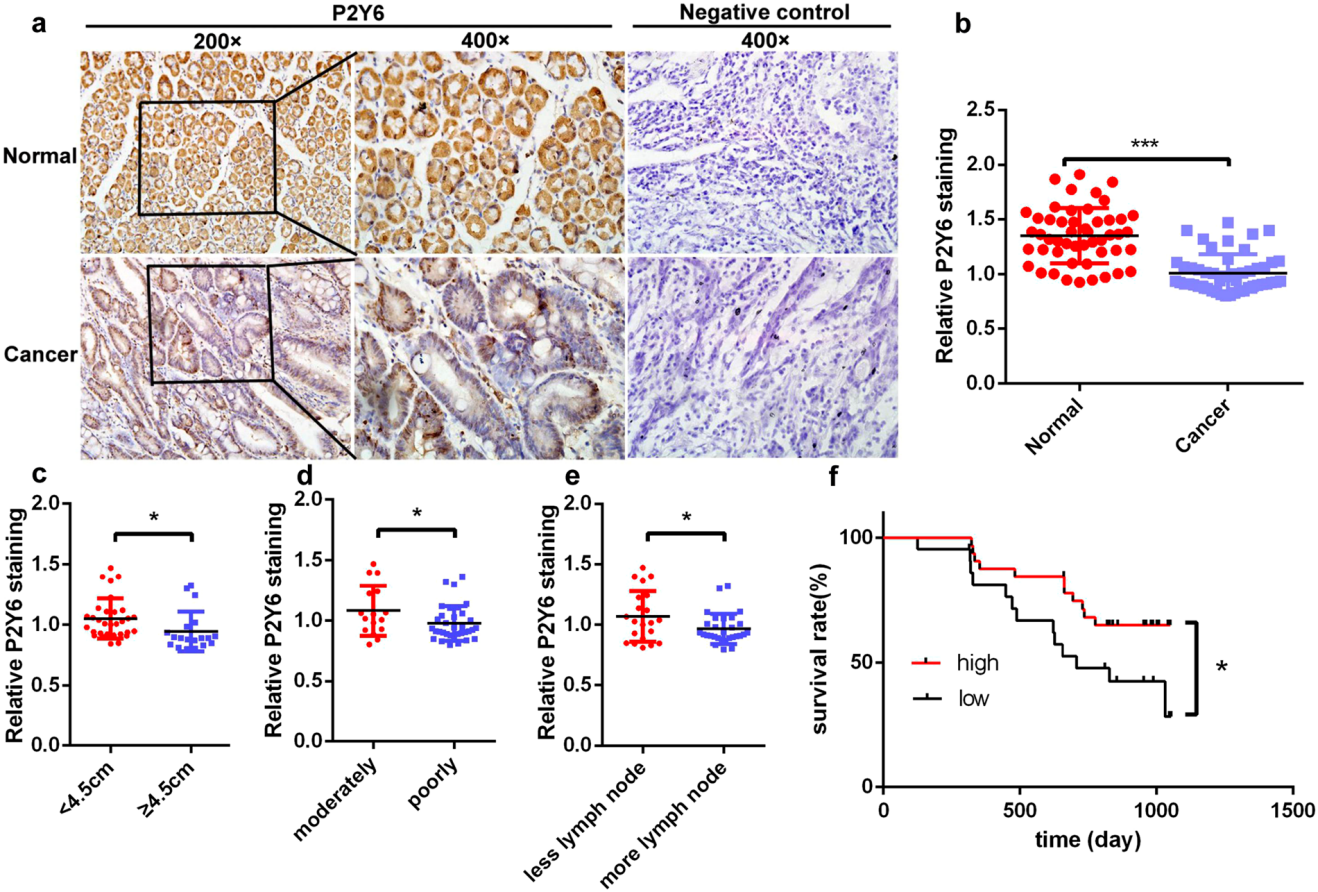
The expression of P2Y6 receptors in human gastric cancer cells and its association with cancer progression. (**a**) Immunohistochemical staining of P2Y6 receptor expression in human GC tissues and adjacent normal tissues. (**b**) Summary data showing the reduced expression of P2Y6 receptors in GC tissues compared to adjacent normal tissues. (**c**–**e**) The expression levels of P2Y6 receptors in GC samples from the patients with different tumor sizes, differentiation and metastasis to lymph nodes. (**f**) Kaplan-Meier analysis of different expression levels of P2Y6 receptors with the survival of GC patients. *p < 0.05, ***p < 0.001, n = 50. Image-Pro Plus 6.0 was used to analysis the P2Y6 receptor expression of immunohistochemical staining.

We first examined the protein expression levels of P2Y6 receptors in human primary GC tissues, and then compared them with those in the normal tissues. Representative immunohistological staining revealed that GC tissues exhibited little positive immunostaining of P2Y6 receptors, but strong staining in normal gastric tissues (Fig. 1a). As shown in the summary data of Fig. 1b, the protein expression levels of P2Y6 receptors were significantly lower in human GC tissues compared to normal tissues. We next characterized the expression of P2Y2, 4, 6 receptors in human gastric normal epithelial cell line (GES-1) and GC cell lines (SGC-7901 and MKN-45 etc). We found that the expression levels of P2Y2 and P2Y4 receptors were significantly higher in GC cells compared to GES-1 (Supplementary Figure S1a,b). However, consistently with the results obtained from human primary GC tissues, the expression of P2Y6 receptors at both levels of transcripts (Supplementary Figure S1c) and proteins (Supplementary Figure S1d) were dramatically lower in GC cells relative to GES-1. Therefore, while the expression of P2Y2 and P2Y4 receptors is up-regulated in GC cells, and the expression of P2Y6 receptors is down-regulated in primary GC tissues and GC cells, suggesting a possible role of P2Y6 receptors as a tumor suppressor in human GC development.

### Low P2Y6 expression correlates with poor progression and short survival of GC patients

If P2Y6 receptors serve as a tumor suppressor in human GC, their expression levels would be correlated with GC progression and patient survival. We therefore compared expression levels of P2Y6 receptor proteins in primary GC tissues at different ages and genders of GC patients and in the cancer with different sizes, differentiations, stages, metastasis to lymph nodes and distant places and invasive depth. Our data showed that low expression of P2Y6 receptors in GC tissues was associated with big tumor size (Fig. 1c), poor differentiation (Fig. 1d), and more metastasis to lymph nodes (Fig. 1e). However, we did not find any significant changes in P2Y6 expression among other variables, such as patient ages and genders, different stages of GC, metastasis to distant places, and invasive depth (Supplementary Figure S1e). After performing correlation analysis between P2Y6 expression and survival of GC patients, we found high P2Y6 expression in GC with long survival of the patients, but low P2Y6 expression with short survival (Fig. 1f), indicating that P2Y6 receptors serve as a tumor suppressor in human GC.

### Nucleotides repress GC cell proliferation through P2Y6-mediated Ca^2+^ and β-catenin signaling

**Figure 2:**
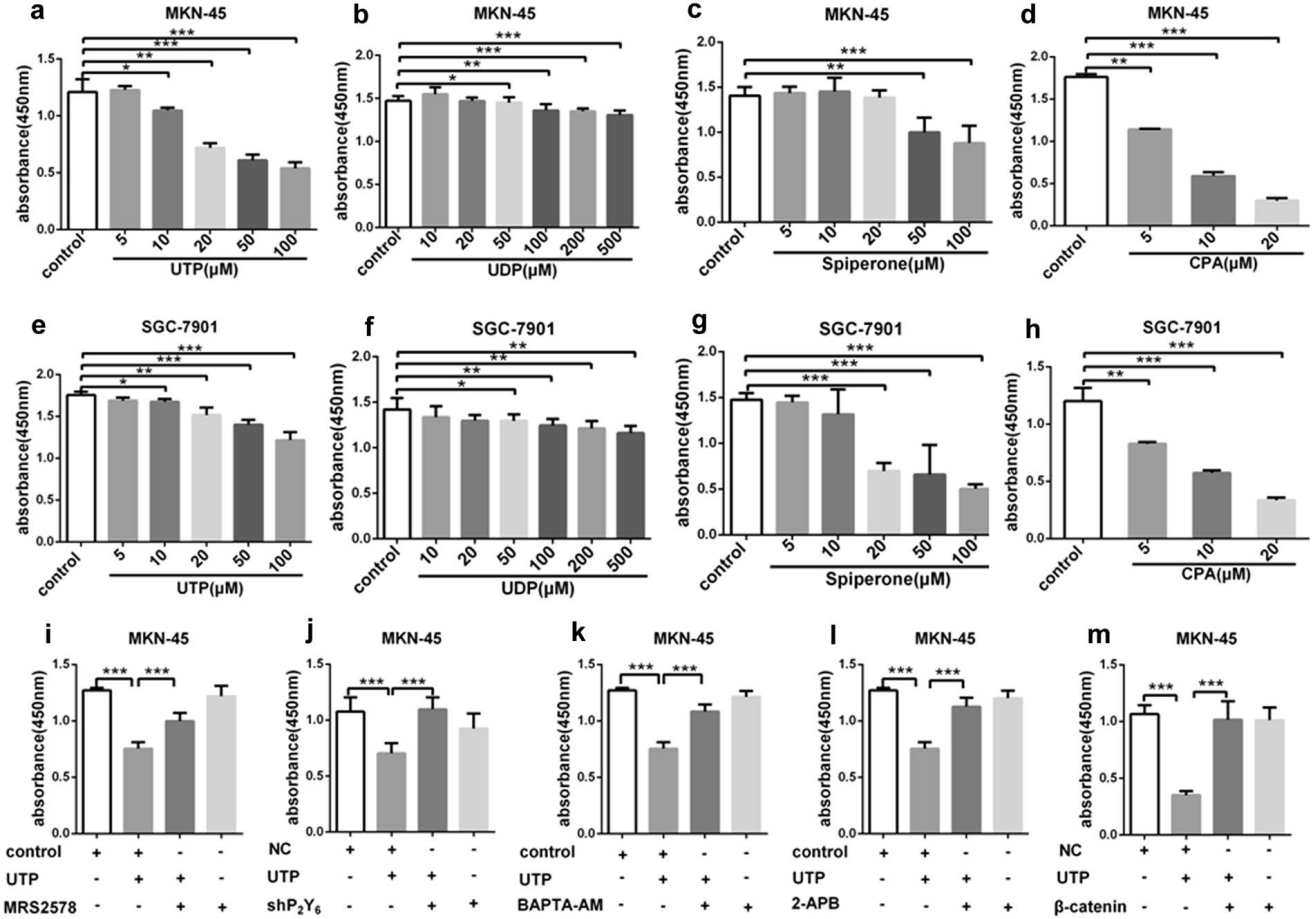
UTP-repressed gastric cancer cell proliferation through P2Y6-mediated Ca^2+^ and β-catenin signaling. (**a**–**h**) UTP, UDP, spiperone and CPA does-dependently inhibited proliferation of MKN-45 and SGC-7901 GC cells. (**i**,**j**) Both P2Y6 receptor selective inhibitor MRS2578 (3 µM) and knockdown of P2Y6 receptors reversed UTP (20 µM)-repressed MKN-45 cell proliferation. (**k**,**l**) Both intracellular calcium chelater BAPTA-AM (1 µM) and SOC inhibitor 2-APB (50 µM) rescued UTP-repressed MKN-45 cell proliferation. (**m**) Over-expression β-catenin rescued UTP-repressed MKN-45 cell proliferation. *p < 0.05, **p < 0.01, ***p < 0.001, n = 3.

We performed cell proliferation to evaluate if activation of P2Y6 receptors really suppresses cancer cell growth. As shown in Fig. 2a,e and b,f, both UTP and UDP dose-dependently inhibited proliferation of cancer cells but not of normal GES-1 cells (Supplementary Figure S4a,b). However, UTP strongly suppressed proliferation of cancer cells with the over-expression of P2Y6 receptors (Supplementary Figure S4c,d). In addition, UTP-repressed MKN-45 cell proliferation could be reversed by either P2Y6 receptor selective inhibitor MRS2578 or knockdown of P2Y6 receptors (Fig. 2i,j). We also showed that the expression of P2Y6 receptors at both levels of mRNA and protein in GC cells were significantly reduced in P2Y6 knockdown group (Supplementary Figure S5a,b,c,d).

Since nucleotides suppress cell proliferation possibly through intracellular Ca^2+^ releasing and then SOC mechanism, we used spiperone^25^, a intracellular Ca^2+^ releasing agent, and cyclopiazonic acid (CPA), a specific SERCA inhibitor that releases intracellular Ca^2+^ as well. Like nucleotides, both spiperone and CPA dose-dependently inhibited GC cell proliferation (Fig. 2c,g,d,h). However, either BAPTA-AM, an intracellular calcium chelator, or 2-aminoethoxydiphenyl borate (2-APB), an inhibitor of both IP_3_ receptor and SOC mechanism^26^, rescued UTP-repressed GC cell proliferation (Fig. 2k,l). Previous studies showed that anti-proliferation of cancer cells induced by Gq/Ca^2+^ signaling pathway could promote nuclear export of β-catenin into cytoplasm where it was degraded by calpain in a Ca^2+^-dependent manner^27^. We also revealed that over-expression of β-catenin could rescue UTP-repressed GC cell proliferation (Fig. 2m and Supplementary Figure S4e,f). Collectively, these data suggest that P2Y6 receptors may repress β-catenin-mediated GC cell proliferation through intracellular Ca^2+^ signaling, which will be further studied in detail in the following studies.

### Nucleotides block the G1/S transition through P2Y6-mediated Ca^2+^ signaling

**Figure 3:**
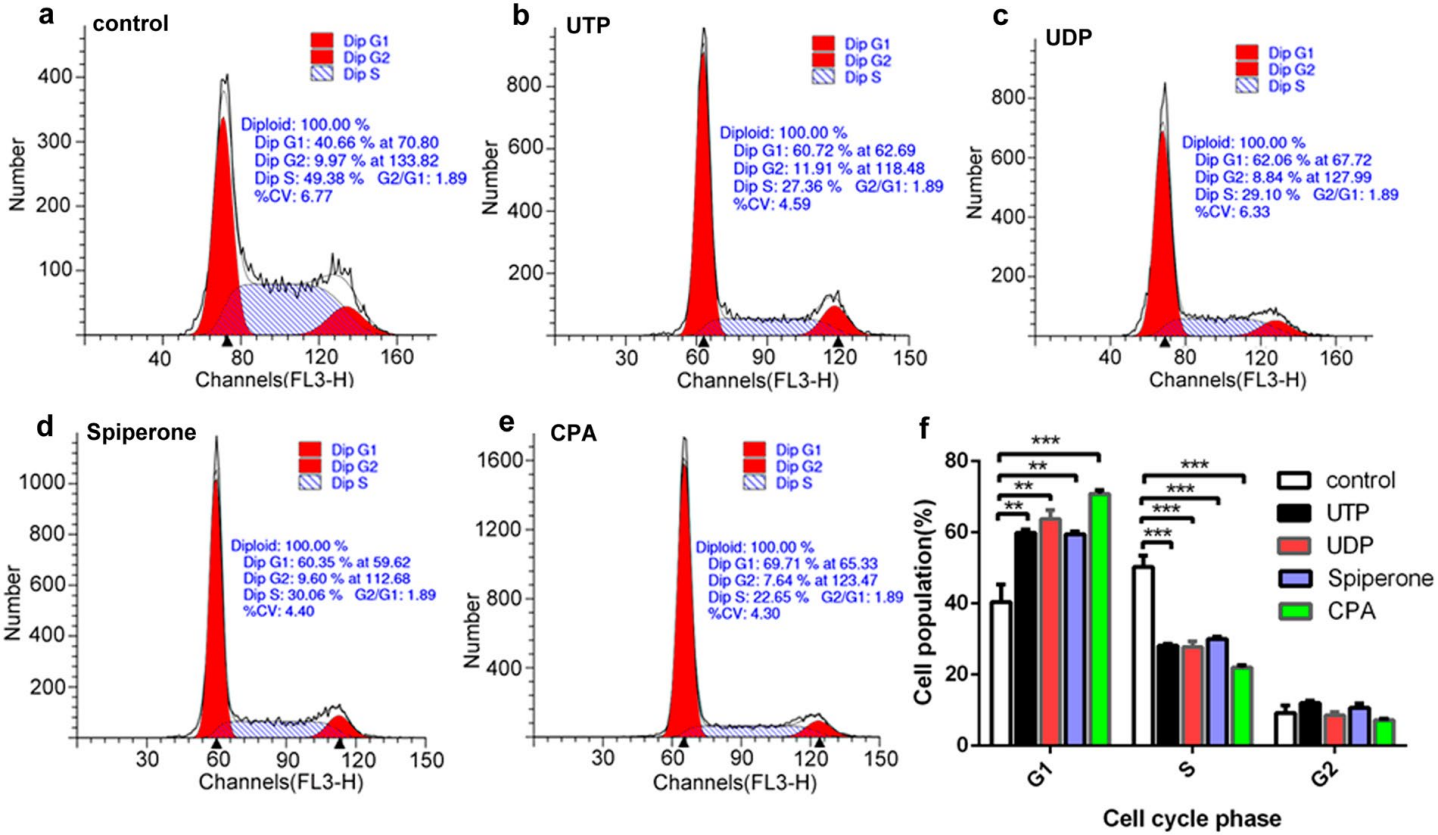
Effects of UTP, UDP, spiperone and CPA on cell cycles of SGC-7901 cells. (**a**–**e**) UTP (20 µM), UDP (50 µM), spiperone (10 µM) and CPA (10 µM) caused G1 phase arrest at different levels and reduced cell population in S phase without affecting G2 phase of the cell cycle. (**f**) Summary data of percentage of cell population in G1, S and G2 phase. **p < 0.01, ***p < 0.001, n = 3.

We determined whether UTP inhibits cell proliferation or induces cell apoptosis. Cell cycle analysis revealed that UTP markedly caused G1 phase arrest (Fig. 3a,b), and flow cytometry analysis indicated that UTP did not influence cell apoptosis (Supplementary Figure S2a,b). Similarly, UDP, spiperone and CPA also caused G1 phase arrest at different levels (Fig. 3c,d,e). As shown in summary data of Fig. 3f, all agents exhibited G1 phase arrest and reduced cell population in S phase without affecting G2 phase of the cell cycle, but they did not influence cell apoptosis (Supplementary Figure S2c,d,e). Moreover, neither UTP nor spiperone affected the expression of caspase-3, a critical apoptosis protease in GC cells (Supplementary Figure S2f,g). These data indicate that UTP does not induce apoptosis but particularly blocks the G1/S transition of GC cells likely through P2Y6-mediated intracellular Ca^2+^ signaling.

### Nucleotides attenuate cyclin D1 and ppRb through P2Y6-mediated Ca^2+^ and β-catenin signaling

**Figure 4:**
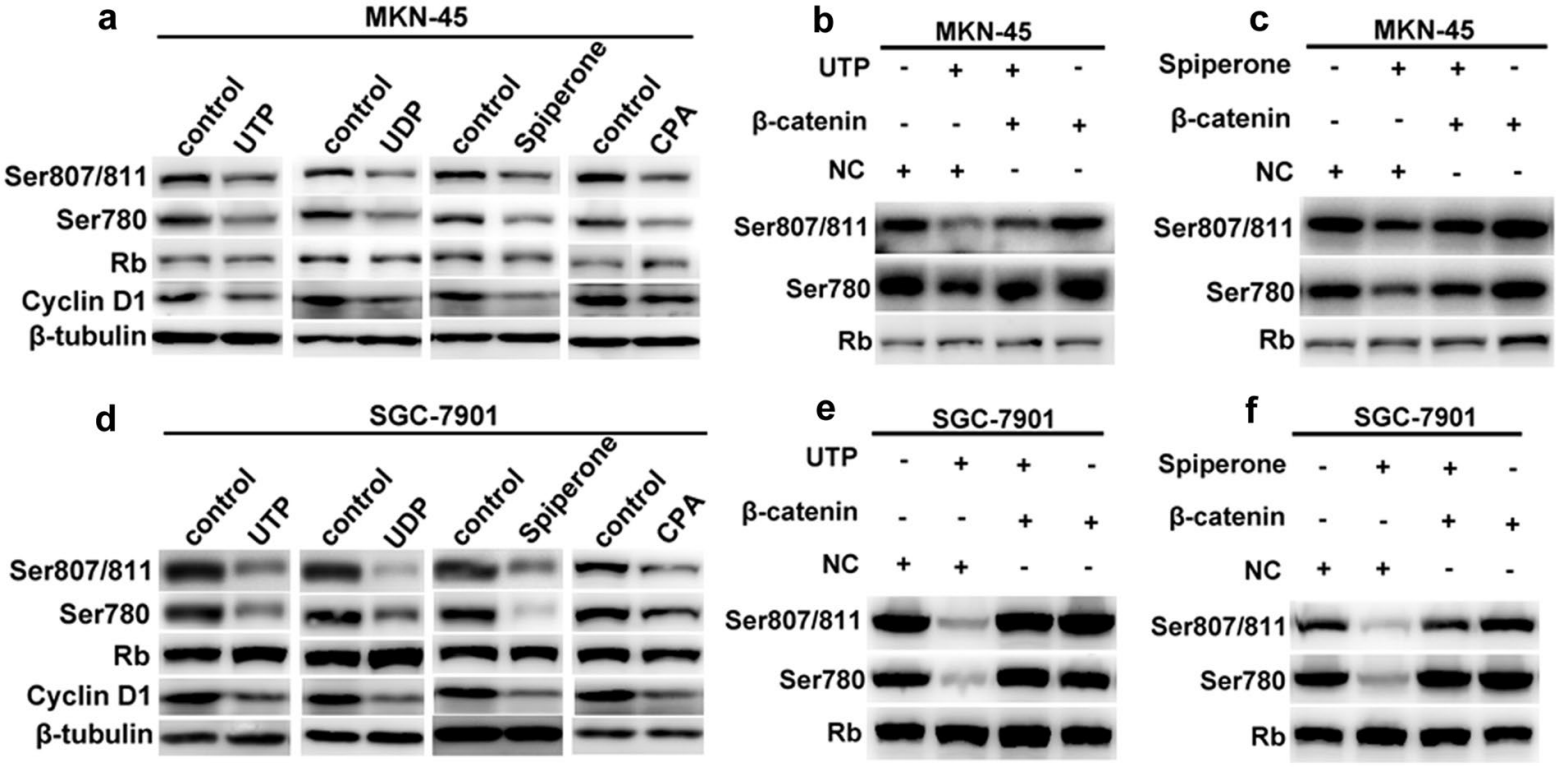
UTP-attenuated cyclin D1 and ppRb through P2Y6-mediated Ca^2+^ and β-catenin signaling. (**a** and **d**) UTP (20 µM), UDP (50 µM), spiperone (10 µM) and CPA (10 µM) repressed cyclin D1 and ppRb (Ser807/811&Ser780) in MKN-45 and SGC-7901 cells. (**b** and **e**) Over-expression β-catenin rescued UTP-repressed cyclin D1 and ppRb (Ser807/811&Ser780) in MKN-45 and SGC-7901 cells. (**c** and **f**) Over-expression β-catenin rescued spiperone-repressed cyclin D1 and ppRb (Ser807/811 & Ser780) in MKN-45 and SGC-7901 cells.

It is well known that cyclin D1 initiates the phosphorylation of pRb (ppRb), which in turn results in pRb inactivation and the release of E2F that consequently transactivates the genes required for S-phase entry^28^. Because P2Y6-mediated Ca^2+^ signaling blocks G1/S transition, we tested whether they could influence these events. As expected, we found that UTP, UDP, spiperone and CPA all repressed cyclin D1 and ppRb (Ser807/811 & Ser780) in GC cells (Fig. 4a,d), suggesting that P2Y6 activation blocks G1/S transition likely through Ca^2+^ **-**dependent suppression of cyclin D1 and ppRb. To further test if β-catenin signaling is involved these events, we over-expressed β-catenin and revealed it could rescue UTP-repressed ppRb (Ser807/811 & Ser780) in GC cells (Fig. 4b,e). Similarly, over-expression of β-catenin could also rescue spiperone-repressed ppRb (Ser807/811 & Ser780) (Fig. 4c,f). Collectively, these data suggest that P2Y6 activation suppresses cyclin D1 and ppRb to block G1/S transition in GC cells likely through Ca^2+^ **/**β-catenin signaling.

### P2Y6 mediates nucleotides-induced Ca^2+^ signaling

**Figure 5:**
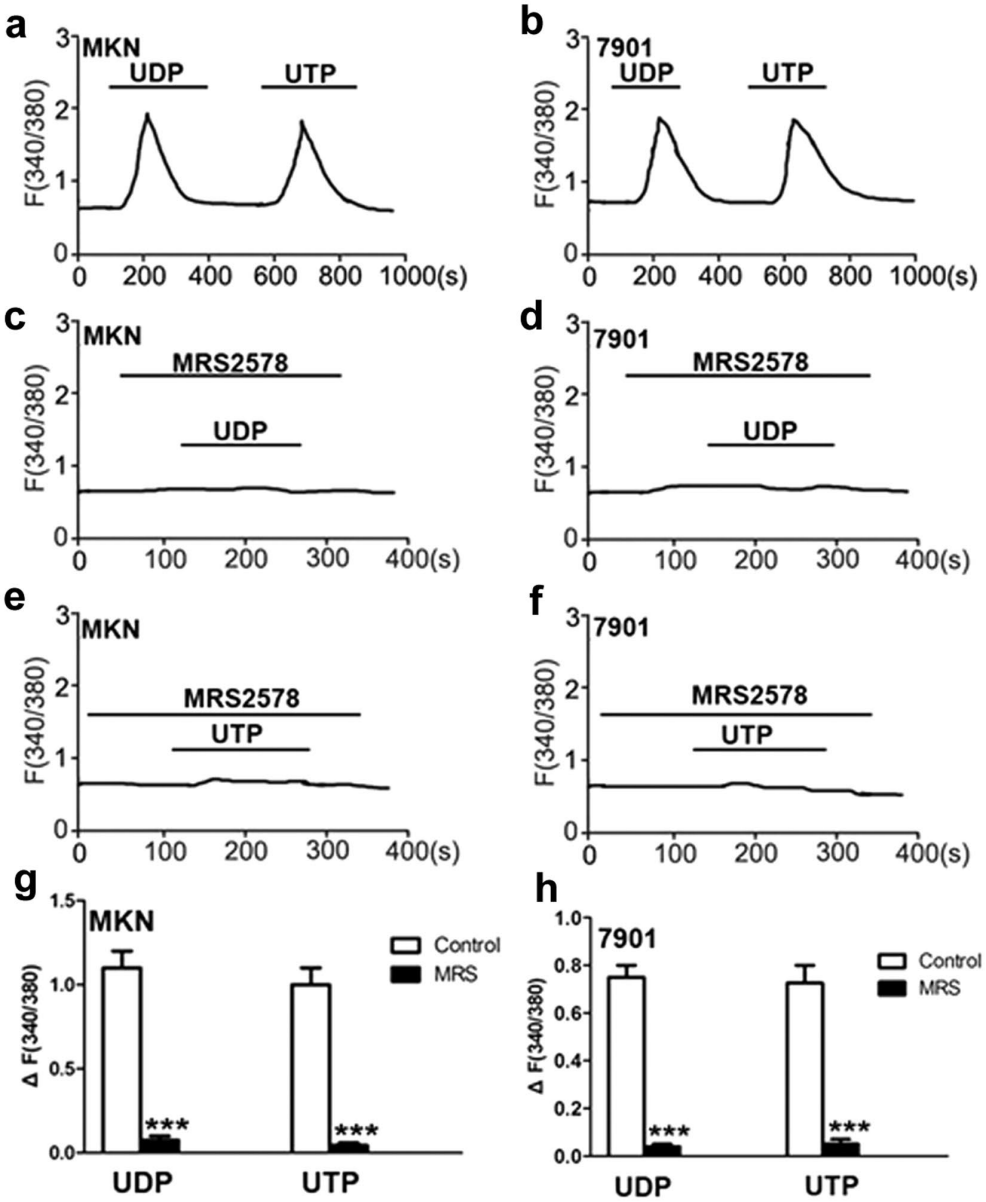
Nucleotides-induced Ca^2+^ signaling through activation of P2Y6 receptors in gastric cancer cells. (**a**,**b**) Time courses of [Ca^2+^]_cyt_ changes induced by UDP (50 µM) and UTP (20 µM) in MKN-45 cells (**a**) and SGC-7901 cells (**b**). (**c**,**d**) Effect of MRS2578 (10 µM) on UDP-induced [Ca^2+^]_cyt_ in MKN-45 cells (**c**) and SGC-7901 cells (**d**). (**e**,**f**) Effect of MRS2578 (10 µM) on UTP-induced [Ca^2+^]_cyt_ in MKN-45 cells (**e**) and SGC-7901 cells (**f**). (**g**,**h)** Summary data showing the [Ca^2+^]_cyt_ changes induced by nucleotides and effect of MRS2578 on nucleotides-induced [Ca^2+^]_cyt_ in MKN-45 cells (**g**) and SGC-7901 cells (**h**). Data are shown as the mean ± SD of 40–50 cells for each group, ***P < 0.001 vs control.

Since nucleotides-mediated [Ca^2+^]_cyt_ plays an important role in cancer development^29–31^, we examined UTP-induced Ca^2+^ signaling in GC cells. Our results showed that UTP at 10 µM stimulated [Ca^2+^]_cyt_ increase in GC cells (Fig. 5a,b). As nucleotides are able to activate P2Y receptors, it is reasonable to speculate that UTP may activate P2Y6 receptors to raise [Ca^2+^]_cyt_. To test this notion, we applied UDP and MRS2578, a selective agonist and antagonist of P2Y6 receptors. As expected, UDP at 10 µM, induced a similar magnitude of [Ca^2+^]_cyt_ increase in GC cells (Fig. 5a,b). Moreover, MRS2578 inhibited both UTP- and UDP-induced [Ca^2+^]_cyt_ increases in GC cells (Fig. 5c,d,e,f, g,h). These data strongly suggest that P2Y6 receptors mainly contribute to UTP-induced [Ca^2+^]_cyt_ increase in GC cells.

### UTP induces Ca^2+^ signaling through SOC mechanism

**Figure 6:**
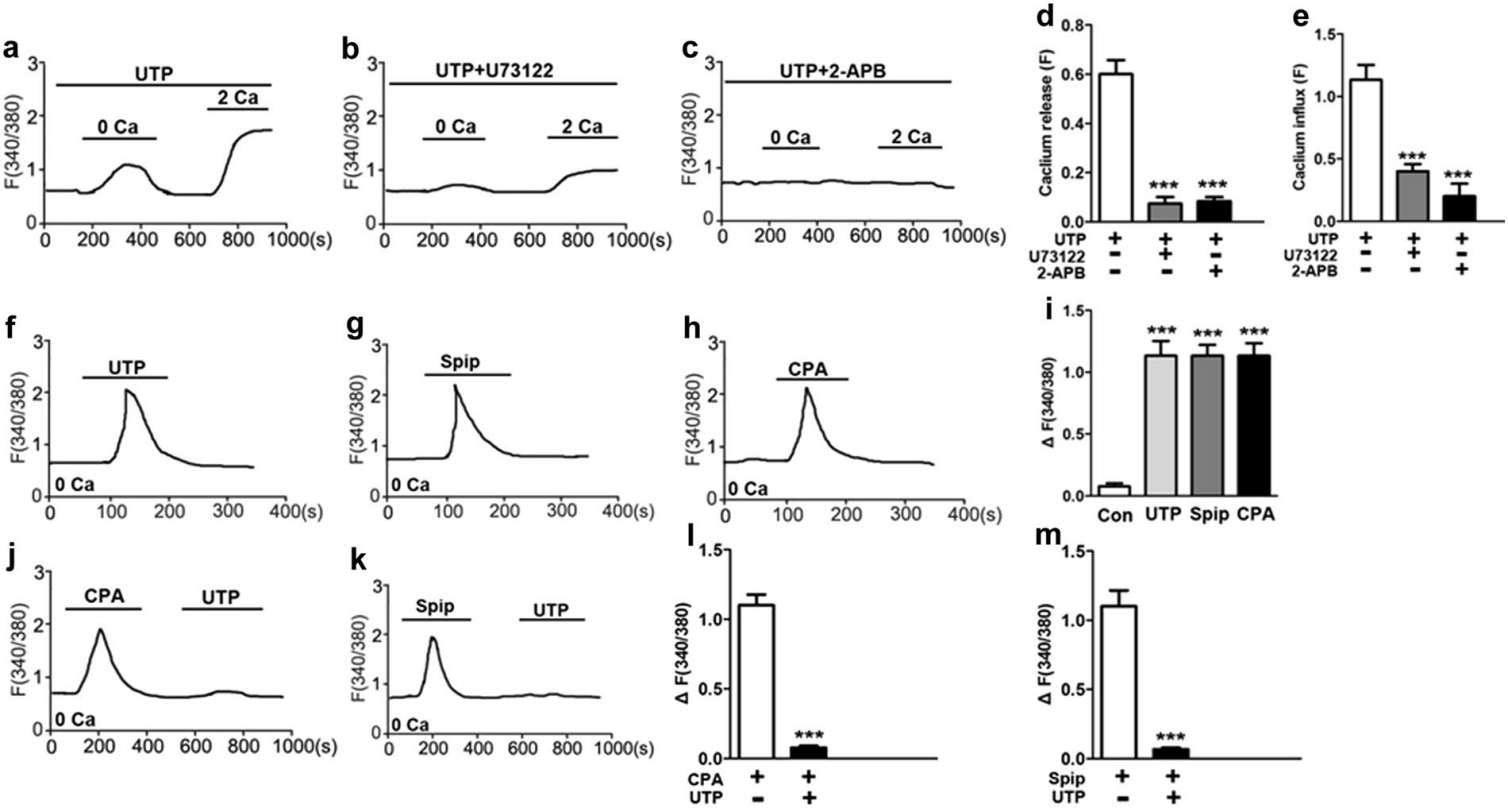
UTP-induced Ca^2+^ signaling through SOC mechanism in gastric cancer cells. (**a**) Time courses of [Ca^2+^]_cyt_ changes induced by UTP (20 µM) in the absence or the presence of extracellular Ca^2+^ in MKN-45 cells. (**b**,**c**) Effect of U73122 (10 µM) or 2-APB (50 µM) on UTP-induced [Ca^2+^]_cyt_ changes in MKN-45 cells. (**d**,**e**) Summary data showing the effect of U73122 or 2-APB on UTP-induced [Ca^2+^]_cyt_ in MKN-45 cells (**d**) and SGC-7901 cells (**e**). (**f**–**h**) Time courses of [Ca^2+^]_cyt_ changes induced by UTP, siperone (10 µM) or CPA (10 µM) in the absence of extracellular Ca^2+^ in MKN-45 cells. (**i**) Summary data showing UTP, siperone or CPA induced [Ca^2+^]_cyt_ in the absence of extracellular Ca^2+^. (**j**,**k**) time courses of [Ca^2+^]_cyt_ changes induced by CPA and UTP, or siperone and UTP in the absence of extracellular Ca^2+^ in MKN-45 cells. (**l**,**m**) Summary data showing CPA and UTP, or siperone and UTP induced [Ca^2+^]_cyt_ in the absence of extracellular Ca^2+^. Data are shown as the mean ± SD of 40–50 cells for each group, ***P < 0.001 vs control.

We further studied the underlying mechanisms of UTP-induced Ca^2+^ signaling in GC cells. We revealed that UTP induced an initial [Ca^2+^]_cyt_ increase in Ca^2+^-free medium, and then a sustained [Ca^2+^]_cyt_ increase in Ca^2+^-containing medium in MKN-45 cells (Fig. 6a,d,e), suggesting UTP causes both intracellular Ca^2+^ release and extracellular Ca^2+^ influx. Further results showed that either U73122, a phospholipase C inhibitor, or 2-APB, markedly eliminated UTP-induced Ca^2+^ release and Ca^2+^ influx (Fig. 6b,c,d,e). We also compared UTP with siperone and CPA in terms of their abilities to induce intracellular Ca^2+^ release and found they caused similar Ca^2+^ release in Ca^2+^-free medium (Fig. 6f,g,h,i). In order to further verify UTP-induced intracellular Ca^2+^ release, we first applied CPA or spiperone to deplete intracellular Ca^2+^ store. Afterwards, application of UTP could not cause further Ca^2+^ release (Fig. 6j,k,l,m). These results strongly indicate that UTP induces Ca^2+^ signaling through intracellular Ca^2+^ release and SOCE in GC cells.

### P2Y6-induced Ca^2+^ signaling suppresses β-catenin

**Figure 7:**
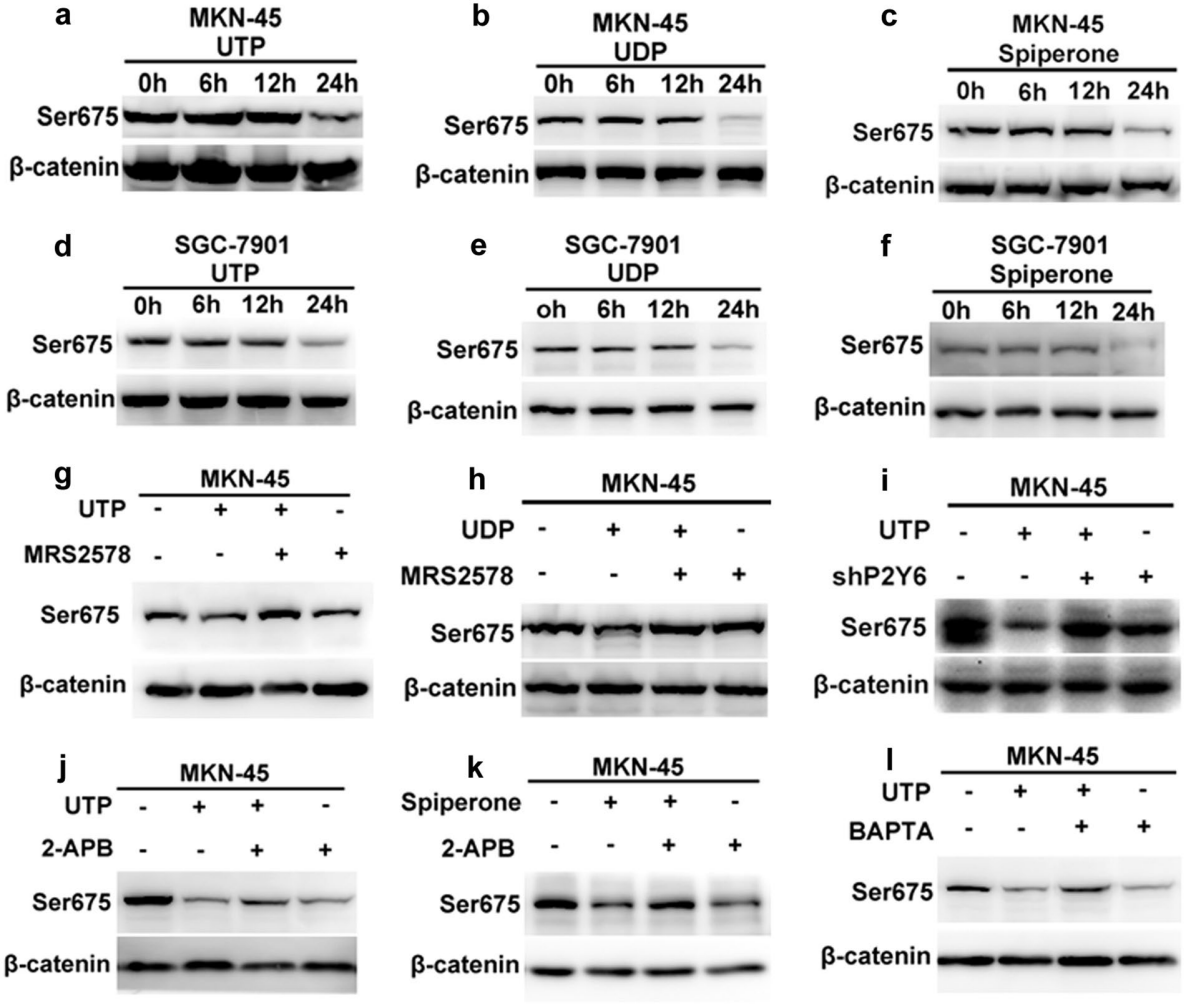
Suppression of β-catenin by P2Y6-induced Ca^2+^ signaling in GC cells. (**a**–**f**) UTP (20 µM), UDP (50 µM) and spiperone (10 µM) decreased β-catenin(Ser675) at 24 hours of MKN-45 and SGC-7901 cells. (**g**,**h**) MRS2578 (3 µM) reversed both UTP and UDP-repressed β-catenin(Ser675). (**i**) Knockdown of P2Y6 receptors reversed UTP induced decrease of β-catenin(Ser675). (**j**,**k**) 2-APB (50 µM) reversed UTP and spiperone-induced decrease of β-catenin(Ser675). (**l**) BAPTA (1 µM) reversed UTP induced decrease of β-catenin(Ser675).

Since it is well documented that β-catenin is critical for cancer cell proliferation, we further studied its role in P2Y6-induced Ca^2+^ signaling in GC cells. We first compared the time course of UTP-induced phosphorylation of β-catenin at Ser-675, an amino acid residue that promotes β-catenin transcriptional activity^32^. β-catenin(Ser675) was significantly decreased when GC cells were treated with UTP for 24 hours (Fig. 7a,d). Similarly, UDP and spiperone also decreased β-catenin(Ser675) (Fig. 7b,c,e,f). Moreover, UTP- and UDP-suppressed phosphorylation of β-catenin(Ser675) was rescued by MRS2578 or knockdown of P2Y6 receptors (Fig. 7g,h,i). Finally, UTP- and spiperone-suppressed phosphorylation of β-catenin(Ser675) was rescued by BAPTA-AM and 2-APB (Fig. 7j,k,l). We also tested if AKT possibly regulates P2Y6-mediated β-catenin signaling, but neither UTP nor UDP could affect AKT phosphorylation (Supplementary Figure S3a,b,c,d). Moreover, we tested the effects of both UTP and UDP on the phosphorylation of NF-κB p65, but did not find any effects (Supplementary Figure S3e,f,g,h). Therefore, β-catenin is specific and critical in P2Y6-induced Ca^2+^ signaling through SOCE in GC cells.

### P2Y6 activation suppresses gastric tumor growth *in vivo*

**Figure 8:**
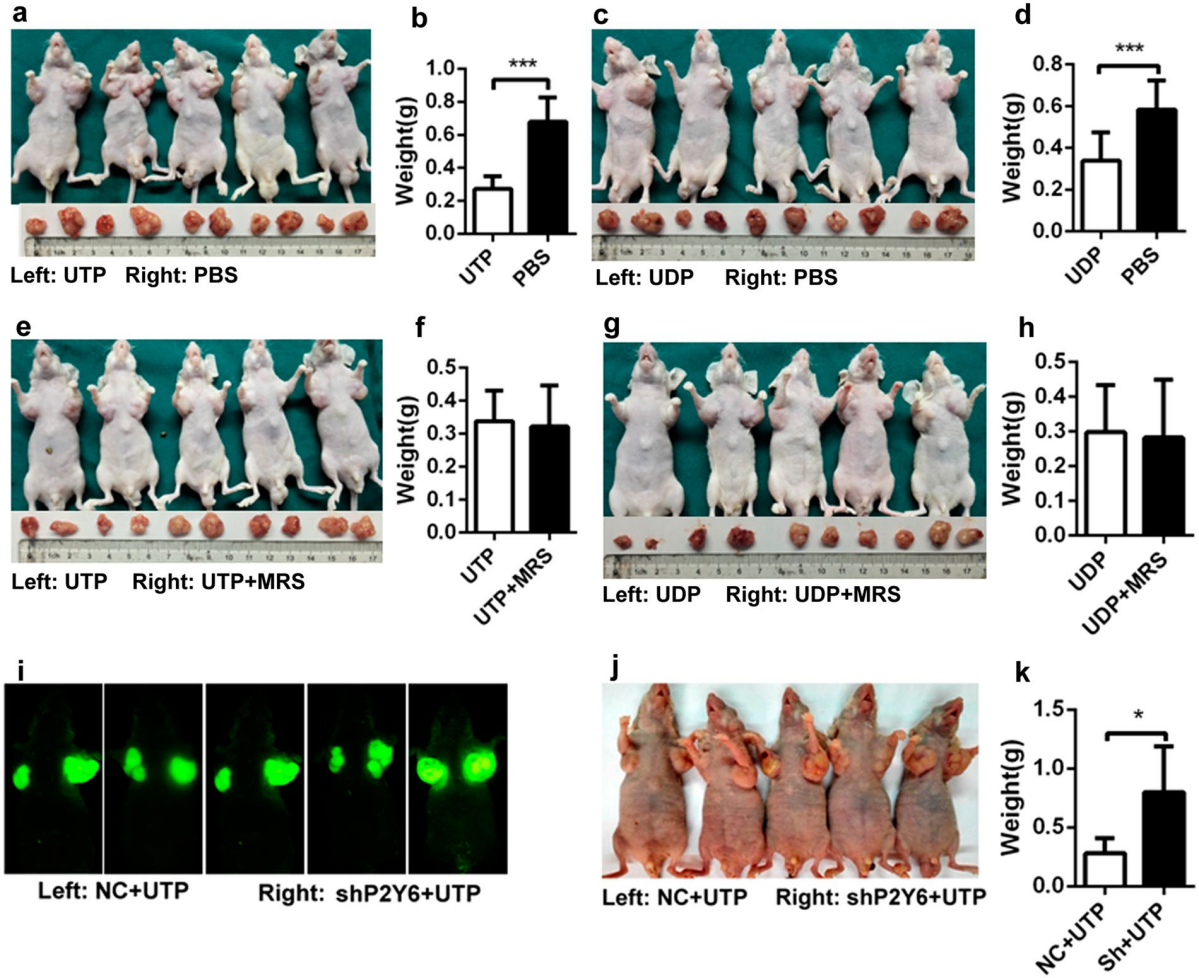
P2Y6 receptors-mediated suppression of gastric tumor growth in mice. (**a**–**h**) Nude mice subcutaneously xenografted in their armpits of SGC-7901 cell. (**a**–**d**) Both UTP (50 µM) and UDP (100 µM, the left side) inhibited tumor weight compared to PBS (the right side). (**e**–**h**) MRS2578 (10 µM, the right side) rescued both UTP and UDP (the left side)-suppressed tumor weight. (**i**–**k**) Nude mice subcutaneously xenografted in their armpits of SGC-7901 cell pretreated with stably transfecting P2Y6 receptors-shRNA (the right side) and P2Y6 receptors-NC lentiviruses (the left side). (**i**) Small animals fluorescence imaging showed knockdown of P2Y6 receptors increased tumor weight. (**j)** White light photographs. (**k**) Summary data of the tumor weight in knockdown of P2Y6 receptors and NC-treated groups. *p < 0.05, ***p < 0.001, n = 5.

To examine if UTP indeed suppresses gastric tumor growth *in vivo*, we established subcutaneously xenografted GC model of nude mice. As shown in Fig. 8a–d, both UTP and UDP decreased the tumor weight in GC group compared to the control group. Moreover, the effects of UTP- and UDP-suppressed tumor were reversed by MRS2578 (Fig. 8e,f,g,h). To provide direct evidence for the role of P2Y6 receptors in gastric tumor growth *in vivo*, their expression in SGC-7901 cancer cells was knocked down by transfecting the cells with P2Y6 receptors-shRNA lentiviruses. However, control SGC-7901 cancer cells were transfected with P2Y6 receptors-NC lentiviruses. The SGC-7901 cancer cells transfected with NC or shRNA against P2Y6 receptors were implanted to the left or right armpits of nude mice, respectively. Afterwards, UTP was injected into each side of armpits to stimulate P2Y6 receptors.

As showed both in image (Fig. 8i,j) and summary data (Fig. 8k), tumor volume and weight were significantly increased in P2Y6 receptors-shRNA group as compared to NC group. Moreover, Western blotting and immunohistochemistry analysis were performed to confirm the effective knockdown of P2Y6 receptors in the SGC-7901 GC cells and tumor tissues. Our data showed that both mRNA and protein expressions of P2Y6 receptors were significantly reduced in the group with the knockdown of P2Y6 receptors compared to the NC group (Supplementary Figure S5a,b), which was further confirmed by immunhistochemistry analysis on the tumor sections obtained from nude mice (Supplementary Figure S5e). In contrast, expression of PCNA and Ki-67, two well-known markers of cellular proliferation, was significantly increased in the group with the knockdown of P2Y6 receptors compared to NC group (Supplementary Figure S5e). Collectively, these results confirm that P2Y6 receptors play a major role in nucleotides-suppressed gastric tumor growth *in vivo*.

## Discussion

In the present study, we demonstrate for the first time that: (1) while multiple subtypes of P2Y receptors are expressed in human gastric epithelial cells, the expression P2Y2 and P2Y4 receptors are up-regulated in GC cells; (2) however, the expression of P2Y6 receptors is down-regulated in both human primary GC tissues and cancer cells; (3) the expression levels of P2Y6 receptors correlate with GC progression and survival of GC patients; (4) nucleotides suppress GC cell proliferation and tumor growth through a novel P2Y6/SOCE/Ca^2+^ relay-mediated inhibition of β-catenin; (5) SOCE/Ca^2+^-mediated anti-proliferation in GC is different from their canonical apoptotic effects in other types of tumors.

Nucleotides are ubiquitous extracellular signaling molecules that induce a wide spectrum of biological responses. Although P2Y receptors are abundantly expressed and biologically important in the myenteric neurons of human digestive system, their expression and function in gastric epithelial cells are poorly understood, and especially their implications in GC development are currently unknown. We therefore investigated the expression and involvement of P2Y receptor subtypes in GC, which is an important and unclear issues. By performing PCR and Western blot analysis, we revealed the expression of P2Y2, P2Y4 and P2Y6 multiple subtypes in gastric epithelial cells, which is consistent with their well-known functional expression in the myenteric neurons^33^.

ATP and UTP are known to regulate cancer growth in various tumor models, and the previous studies on the role of P2Y receptors in gastrointestinal cancer mainly focused on colorectal cancer^34, 35^. P2Y2 and P2Y4 receptors were found to up-regulated in human colon cancer tissues and cells to have oncogenic potential^36^. Little is known about the role of P2Y6 receptors in colon cancers since only one paper reported that nitric oxide can activate cGMP/PKG pathway to induce apoptosis of SW480 colon cancer cells likely through P2Y6 receptors^37^. Our data show that P2Y6 receptors are down-regulated in primary GC tissues and cells, and that low expression of P2Y6 receptors correlates with big tumor size, poor differentiation, more metastasis to lymph nodes and short survival of GC patients. These results strongly suggest P2Y6 receptors may serve as GC suppressors. In contrast, the up-regulated P2Y2 and P2Y4 receptors suggest their promoting roles in GC as in other GI cancers^38^.

Although UTP can activate three subtypes of P2Y2, P2Y4 and P2Y6 receptors that were all detected in GC cells, UTP attenuated rather than promoted GC cell proliferation, ruling out the possible involvements of P2Y2 and P2Y4 receptors in this process. We therefore concentrated on the role of P2Y6 receptors in GC development and the underlying molecular mechanisms. Indeed, nucleotides did not alter proliferation of normal gastric cells, but suppressed proliferation of GC cells specifically, which could be reversed by MRS2578 and shP2Y6 *in vitro* and *in vivo*. However, nucleotides did not induce apoptosis of GC cells. Taken together, these results strongly support the important anti-proliferative role of P2Y6 receptors in GC cells, which is different their canonical apoptotic role via cGMP/PKG pathway in colon cancer cells^37^.

We further elucidate the molecular mechanisms of P2Y6-mediated GC suppression. Since P2Y activation predominately raises [Ca^2+^]_cyt_ through SOCE mechanisms^31^, we observed both [Ca^2+^]_cyt_ chelator and SOCE blocker reversed UTP-suppressed GC cell proliferation, suggesting the involvements of SOCE/Ca^2+^ in P2Y6-mediated GC suppression. Although the important role of nucleotides-induced Ca^2+^ signaling was previously documented in the development of human cancers^29–31^, little is currently known about its involvement in gastric tumorigenesis. We found that P2Y6 activation raised [Ca^2+^]_cyt_ by inducing intracellular Ca^2+^ release like the well-known ER Ca^2+^ releaser, CPA and spiperone, and in turn Ca^2+^ entry through SOCE mechanisms, all of which could be abolished by inhibitors of PLC and SOCE. It has been reported that SOCE is important in inducting apoptosis of prostate cancer, breast cancer and colon cancer^39, 40^. However, the previous SOCE/Ca^2+^-induced apoptosis is not involved in the P2Y6/SOCE/Ca^2+^-mediated anti-proliferative mechanisms in GC cells. By comparing to CPA and spiperone, we revealed P2Y6/SOCE exhibits anti-proliferation by suppressing cyclin D1 and ppRb to block the G1/S transition in GC cells.

**Figure 9:**
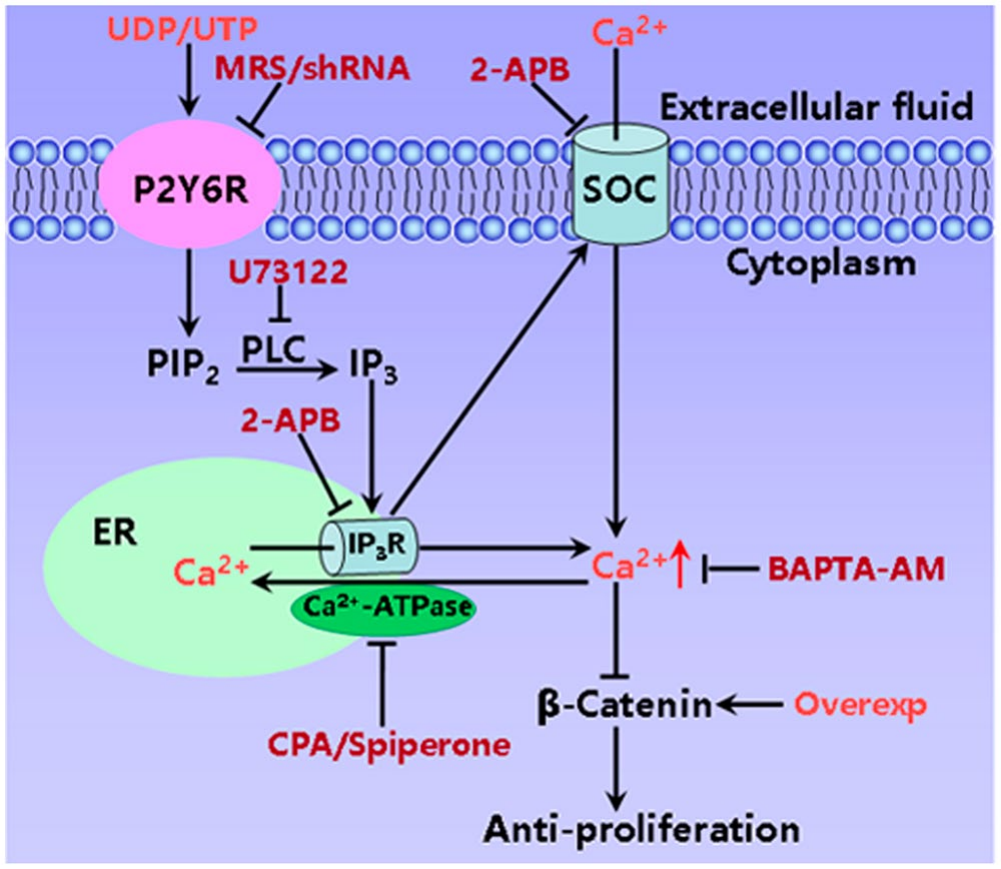
A proposed model for P2Y6-mediated suppression of gastric tumorigenesis through SOCE/Ca^2+^/β-catenin signaling pathways. Activation of P2Y6 receptors induces IP_3_ production by PLC. IP_3_ activates IP_3_R on the ER to induce intracellular Ca^2+^ release and extracellular Ca^2+^ influx via the SOC. Finally Ca^2+^ inhibits β-catenin and suppresses proliferation of GC cells. Overexp: over-expression of β-catenin.

It is known that [Ca^2+^]_cyt_ signaling could stimulate AKT activity that inactivates GSK3β, resulting in the nuclear accumulation of β-catenin, which produces tumorigenesis^41^. Since the regulatory mechanisms of Ca^2+^ signaling on Wnt/β-catenin are poorly understood, we investigated whether P2Y6-mediated Ca^2+^ signaling could modulate β-catenin signaling in GC cells. We did not detect any effects of nucleotides on AKT and NF-κB activity in GC cells, ruling out the involvements of these pathways. However, we found that P2Y6 activation specifically repressed β-catenin phosphorylation through SOCE mechanism in GC cells, which could be reversed by Ca^2+^ chealater and SOC blocker. Parallelly, P2Y6-mediated anti-proliferation could be reversed by SOC blocker and over-expression of β-catenin as well. Therefore, we identified a novel SOCE/Ca^2+^-suppressed β-catenin relay that plays a major role in P2Y6-mediated GC suppression (Fig. 9). Although activation of P2Y6 receptors suppress GC cells through the SOCE/Ca^2+^-dependent pathway, they promote proliferation of pancreatic cancer cells through a Ca^2+^/CaMKII-independent pathway^42^. Thus, P2Y6 receptors may play different roles in GC and pancreatic cancer through various downstream pathways, which is not surprising due to the significant differences in tumorigenesis of the stomach and pancreas.

In conclusion, we demonstrate for the first time that P2Y6 receptors suppress GC growth likely through a novel SOCE/Ca^2+^/β-catenin-mediated anti-proliferation, which is different from the canonical SOCE/Ca^2+^-induced apoptosis in other tumors. Although P2Y6 receptors play different roles in GC and pancreatic cancer, our findings suggest that the P2Y6 receptor-mediated novel downstream pathways might be potential targets for innovative GC prevention/therapy.

## Materials and Methods

### Ethics statement and human tissue samples

All gastric tumor tissues and corresponding adjacent tissues were collected from 50 patients who underwent surgical resection for GC in the Southwest Hospital (during 2010 and 2011) of the Third Military Medical University (TMMU). The diagnosis of GC was further confirmed by the histology of resected specimens. All animal and clinical studies were approved by the Clinical Research Ethics Committee of TMMU. The informed consent was obtained for all patients. All experiments were performed in accordance with the approved guidelines.

### Cell culture and reagents

GES-1 is a human immortalized relative normal gastric epithelial cell line. SGC-7901 and MKN-45 are human gastric cancer cell lines. These cells are accessible to the research community around the world^43–46^. They were obtained from the Type Culture Collection of the Chinese Academy of Sciences. GES-1 and SGC-7901 were maintained in Dulbecco’s modified Eagle’s medium (high glucose) (Hyclone, Logan, UT, USA). MKN-45 cells were cultured in RPMI-1640 medium. All culture medium were supplemented with 10% fetal bovine serum.

Antibodies: Rb, Rb(ser780), Rb(ser807/811), β-catenin, β-catenin(ser675), Caspase-3, CyclinD1, PCNA, Ki67, all were purchased from Cell Signaling Technology (CST). P2Y2, P2Y4 and P2Y6 were obtained from Abcam; β-tubulin was purchased from Santa Cruz Biotechnology. UTP, UDP, spiperone, CPA, MRS2578, U73122, 2-APB, BAPTA/AM, all were purchased from Sigma.

### Immunohistochemistry

The primary antibodies were detected with biotinylated goat anti-rabbit IgG (Vector Laboratories, Burlingame, CA, USA) secondary antibodies. Immunoreactivity was detected using a horseradish peroxidase (3′-, 3′-diaminobenzidine) kit (BioGenex, Fremont, CA, USA), followed by counterstaining with hematoxylin, dehydration and mounting. The section with human GC tissues and corresponding adjacent tissues were incubated with an anti-P2Y6 receptors monoclonal antibody (1:250 dilution, Abcam).

### RNA Extraction and Real-time RT-PCR

Total RNA from GES-1, MKN-45, SGC-7901, BGC-823, AGS, MKN-28, BGC-803 cells; 36 pairs freshly isolated human gastric tumor tissues and corresponding adjacent tissues were extracted using RNAiso Plus reagent (Takara Japan) in accordance with the instructions of the manufacturer. The cDNA was synthesized from 2 µg of total RNA using PrimeScript^®^ RT-polymerase (Takara, Japan) according to the instructions of the manufacturer. Quantitative real-time RT-PCR was performed on Bio-Rad (USA) with SYBR^®^ Premix Ex Taq^TM^ II kit (Takara, Japan). All samples were run in triplicate, and β-tubulin was used as an internal control. The primers were as follows: P2Y6 receptors, AGCAGGAAGCCGATGACAGTGA (forward) and AACCGCACTGTCTGCTATGACC (reverse).

### Western Blot

Total protein was isolated from GES-1, MKN-45, SGC-7901, BGC-823, AGS, MKN-28, BGC-803 lysed by RIPA buffer (Santa Cruz Biotechnology). The protein concentration of the supernatant was measured by BCA protein assay (Pierce). The antibody: P2Y2 (1:1000), P2Y4 (1:1000), P2Y6 (1:10000), Rb (1:1000), Rb(ser780) (1:1000), Rb(ser807/811) (1:1000), β-catenin (1:1000), β-catenin(ser675) (1:1000), Caspase-3 (1:1000), CyclinD1 (1:1000), β-tubulin (1:500). The signals were visualized using enhanced chemiluminescence (ECL, Thermo, USA).

### Cell proliferation assay

Cell proliferation assays were measured using Cell Counting Kit-(CCK-)8 (Dojindo Laboratories, Kumamoto, Japan) according to the instructions of the manufacturer. Each experiment was performed in triplicate and repeated three times.

### Cell cycle analysis

After the SGC-7901 cells were treated by UTP, UDP, spiperone, CPA for 16–24 hours, the cells were harvested and fixed with 4% paraformaldehyde at 4 °C overnight. After washing with staining buffer, cells were incubated in a solution containing 0.2% Tween 20, 100 U/mL RNase, and 50 μg/mL propidium iodide for 20 min at 37 °C. Cell cycle analysis was performed using flow cytometry in which samples were gated on live cells. LMD files were further analyzed using ModFit LT (Verity Software House, Topsham, ME). Each experiment was performed in triplicate and repeated three times.

### Measurement of [Ca^2+^]_cyt_ by digital Ca^2+^ imaging

GC cells cultured on coverslips were loaded with 5 μM Fura-2AM (Invitrogen, NY, USA) in physiological salt solution (PSS) at 22 °C for 50 min and then washed with PSS for 30 min. Then, cells on coverslips were placed in a standard perfusion chamber on the stage of an inverted fluorescence microscope (Nikon, Japan). The ratio of Fura-2 fluorescence with excitation at 340 or 380 nm (F340/380) was followed over time and captured with an intensified CCD camera (ICCD200). Images were acquired using a Meta Fluor software (Universal Imaging Corporation, Downingtown, PA). PSS used in digital Ca^2+^ measurement contained the following (in mM): 140 Na^+^, 5 K^+^, 2 Ca^2+^, 147 Cl^−^, 10 HEPES, and 10 glucose, pH 7.4. For the Ca^2+^-free solution, Ca^2+^ was omitted and 0.5 mM EGTA was added to prevent possible Ca^2+^ contamination.

### Cell transfection

Transfections were performed using Lipofectamine 2000 (Invitrogen, Carlsbad, CA, USA) according to the manufacturer’s instructions. The CTNNB1 (NM_001904.3 humo) was designated as over-expression of β-catenin. To silence P2Y6 receptors, lentivirus-based shRNA was used. shRNA sequences for P2Y6 receptors and control were as follows 5′ to 3′: GCTGAACATCTGTGTCATTAC and TTCTCCGAACGTGTCACGT. SGC-7901 cells were transfected according to the protocol of the manufacturer. The protein expression of P2Y6 receptors was detected by Western blot analysis to demonstrate successful silencing. The CTNNB1, PEX-3 and lentivirus-based shP2Y6 receptors and control were purchased from Genepharma (Shanghai, P.R. China).

### GC xenografts

This study was performed in accordance with the NIH Animal Use Guidelines and a protocol approved by the Animal Care Committee of TMMU. Approximate 1 × 10^8^ SGC-7901 cancer cells were injected into both armpits of 4-week-old male nude mice. After tumor sizes reached about 1 mm^3^, nude mice were divided into the following 4 groups: the tumor on the left or right side was injected with UTP or PBS in group 1, with UDP or PBS in group 2, with UTP or UTP and MRS2578 in group 3, and with UDP or UDP and MRS2578 in group 4, respectively. All injections were once a day for 4 weeks. For knockdowning P2Y6 receptors in GC xenografts, approximate 1 × 10^8^ SGC-7901 cells stably expressing shP2Y6 receptors were injected into the right armpit of 4-week-old male nude mice, while the same amount of SGC-7901 cells containing negative control were injected into the left armpit. After tumor sizes reached about 1 mm^3^, the tumors on both sides were injected with UTP once a day. The mice were euthanized after 4 weeks, and the tumors were visualized with an *In-Vivo* Imaging System (Cambridge Research & Instrumentation, Woburn, MA, USA). The tumors were harvested, weighed and measured.

### Statistical analysis

All data are shown as means ± SE and analyses were performed using Prism 5.0 software (GraphPad, San Diego, CA, USA). The statistical significance between two groups was determined by Student’s t-test. The one-way ANOVA was used to compare three or more groups. Significant difference was expressed in the figures or figure legends as *P < 0.05.

## Electronic supplementary material

Supplementary Information
