# Supplementary material for: Anti-proliferative Effects of Nucleotides on Gastric Cancer via a Novel P2Y6/SOCE/Ca2+/β-catenin Pathway
Source: Sci Rep. 2017 May 26;7:2459. doi: 10.1038/s41598-017-02562-x (PMC5446419; doi:10.1038/s41598-017-02562-x)
Supplement: Supplementary file 1 — Supplementary Information [file 41598_2017_2562_MOESM1_ESM.doc]

**Anti-proliferative Effects of Nucleotides on Gastric Cancer via a Novel P2Y6/SOCE/Ca^2+^/β-catenin Pathway**

Hanxing Wan^1*^, Rui Xie^1*^, Jiangyu Xu^2^, Jialin He^1^, Bo Tang^1^, Qingqing Liu^1^, Sumin Wang^1^, Yanjun Guo^1^, Xin Yang^1^, Tobias Xiao Dong^3^, [John M. Carethers](http://www.sciencedirect.com/science/article/pii/S0304383516302622)^4^, Shiming Yang^1$^ and Hui Dong^1,3$^

**Supplementary figures and figure legends**

**
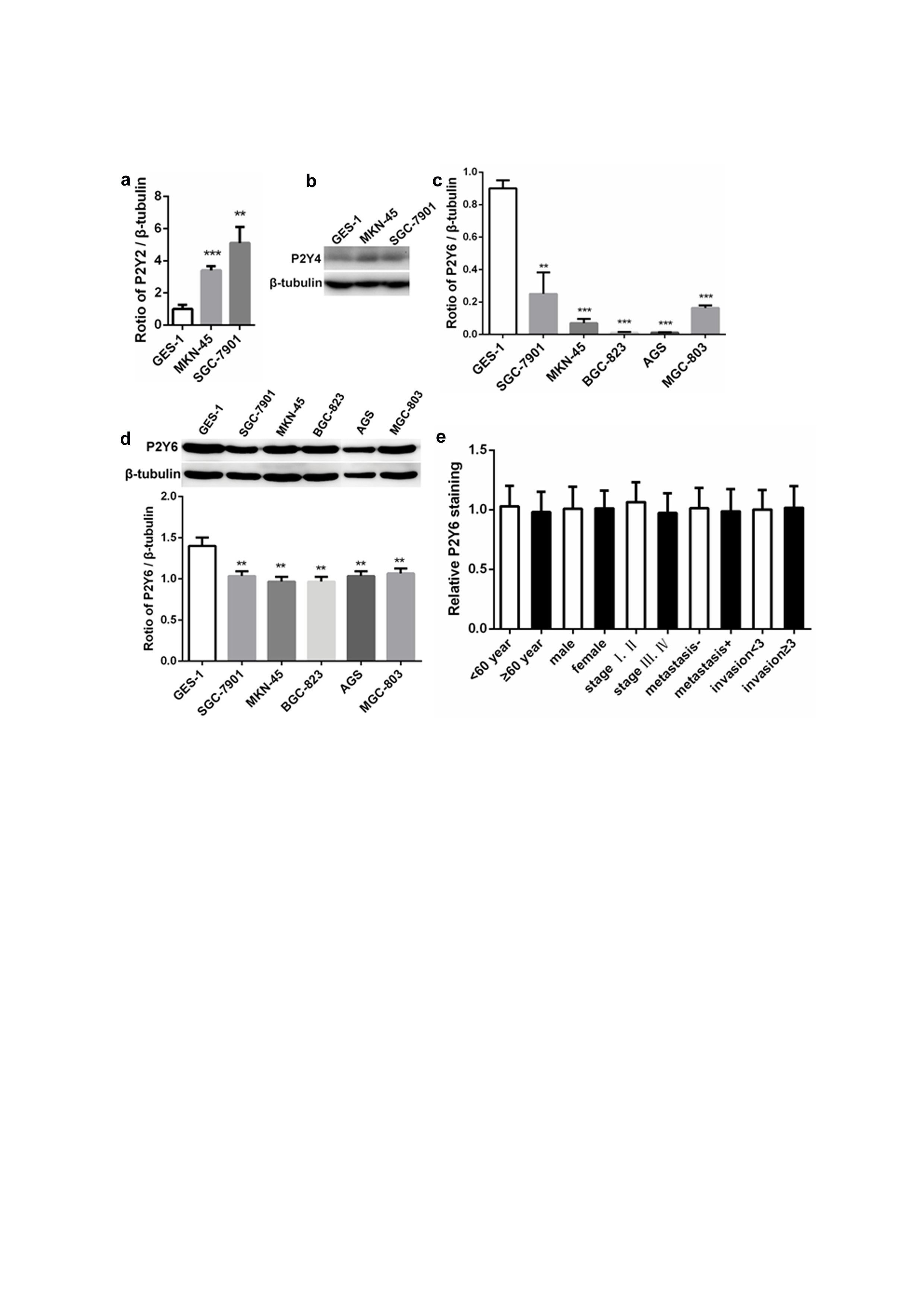
**

**Supplementary figure S1. The expression of different P2Y receptor subtypes in human gastric cell lines and the analysis of P2Y6 receptor expression with different parameters of GC patients. a:** The expression levels of P2Y2 receptor transcripts in human gastric normal epithelial cell line (GES-1) and gastric cancer cell lines (SGC-7901 and MKN-45). **b:** The expression levels of P2Y4 receptor proteins in GES-1, SGC-7901 and MKN-45 cells. **c:** The expression levels of P2Y6 receptor transcripts in GES-1 cells and different gastric cancer cell lines. **d:** The expression levels of P2Y6 receptor proteins in GES-1 cells and different gastric cancer cell lines. **e:** Comparison of P2Y6 receptor expression levels in the GC patients of different ages and genders and in the GC with different stages, metastasis to distant places and invasive depth. No significant difference among all parameters. **p<0.01, ***p<0.001, n=3 for all experiments.

**
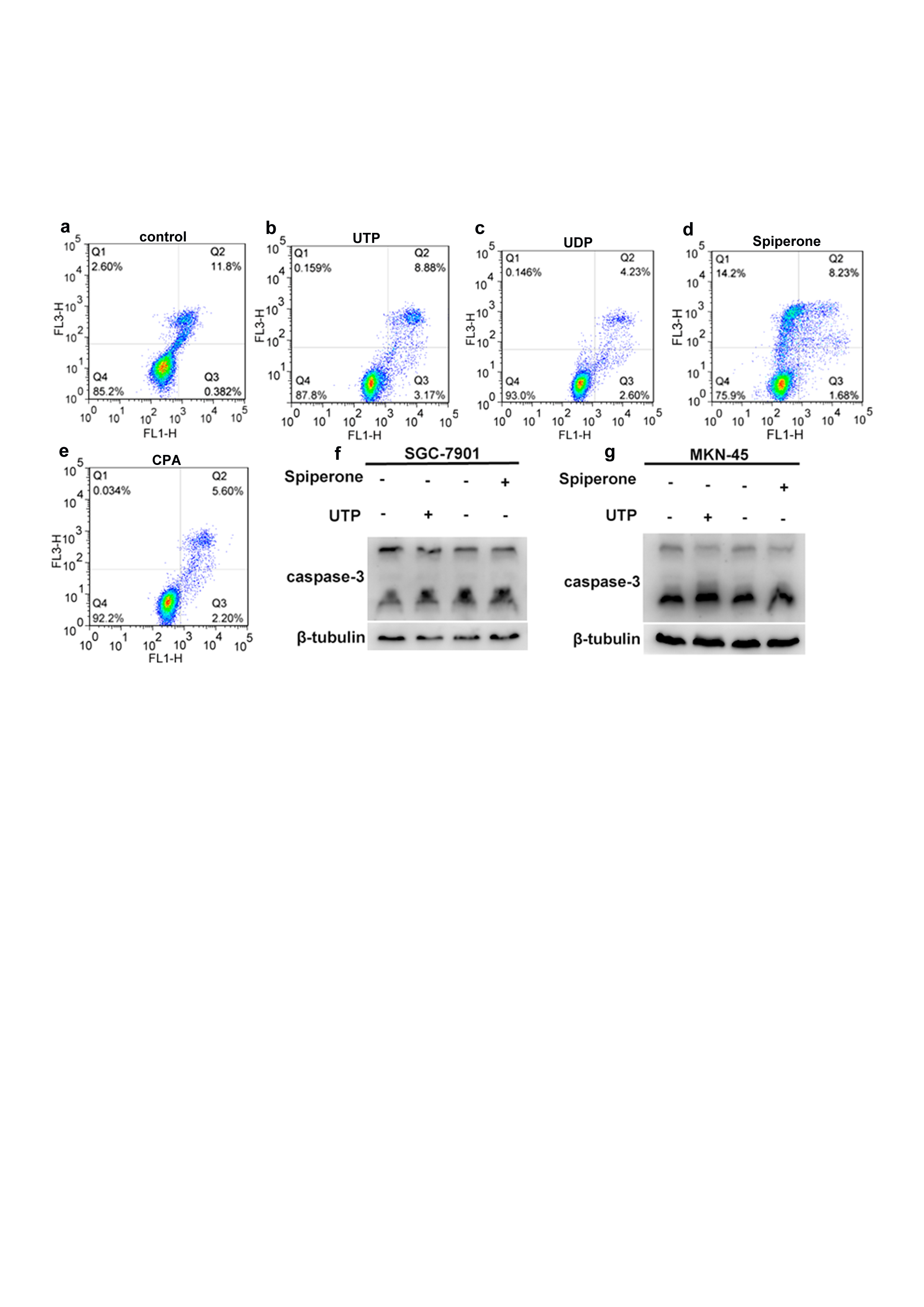
**

**Supplementary figure S2. No influences of nucleotides and intracellular Ca^2+^ releasers on the apoptosis of gastric cancer cells. a-e:** flow cytometry analysis indicates no influences of UTP (20 µM), UDP (50 µM), spiperone (10 µM) and CPA (10 µM) on SGC-7901 cell apoptosis. **f-g:** Western blot reveals no effects of UTP and spiperone on the expression of caspase-3 in both MKN-45 and SGC-7901 cells.


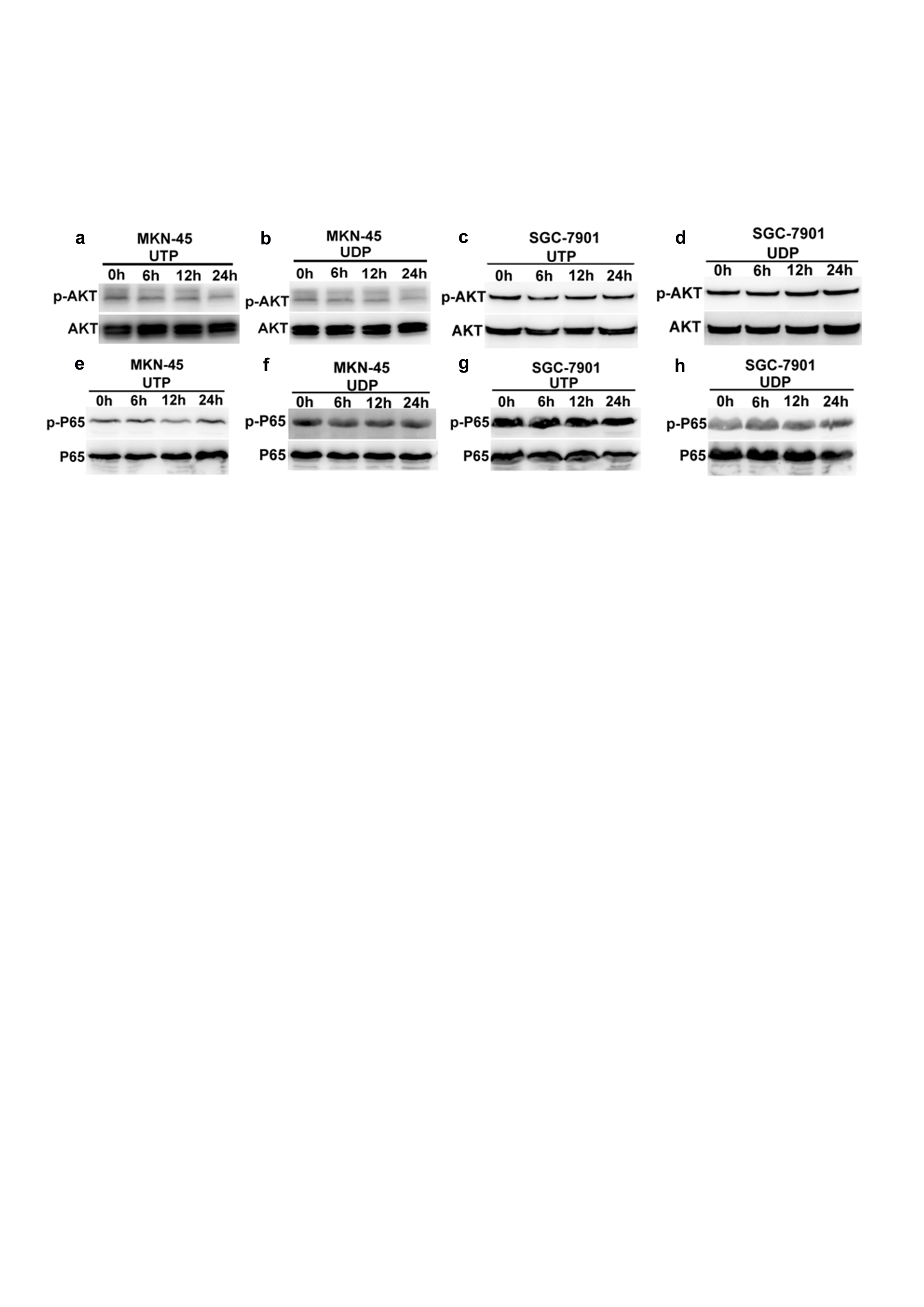


**Supplementary figure S3. Effects of nucleotides and spiperone on phophorylations of AKT and NF-κB in GC cells. a-d:** No effects of UTP (20 µM) and UDP (50 µM) on AKT phophorylation in MKN45 and SGC-7901 cells. **e-h：**No effects of UTP (20 µM) and UDP (50 µM) on NF-κB p65 phosphorylation in GC cells.


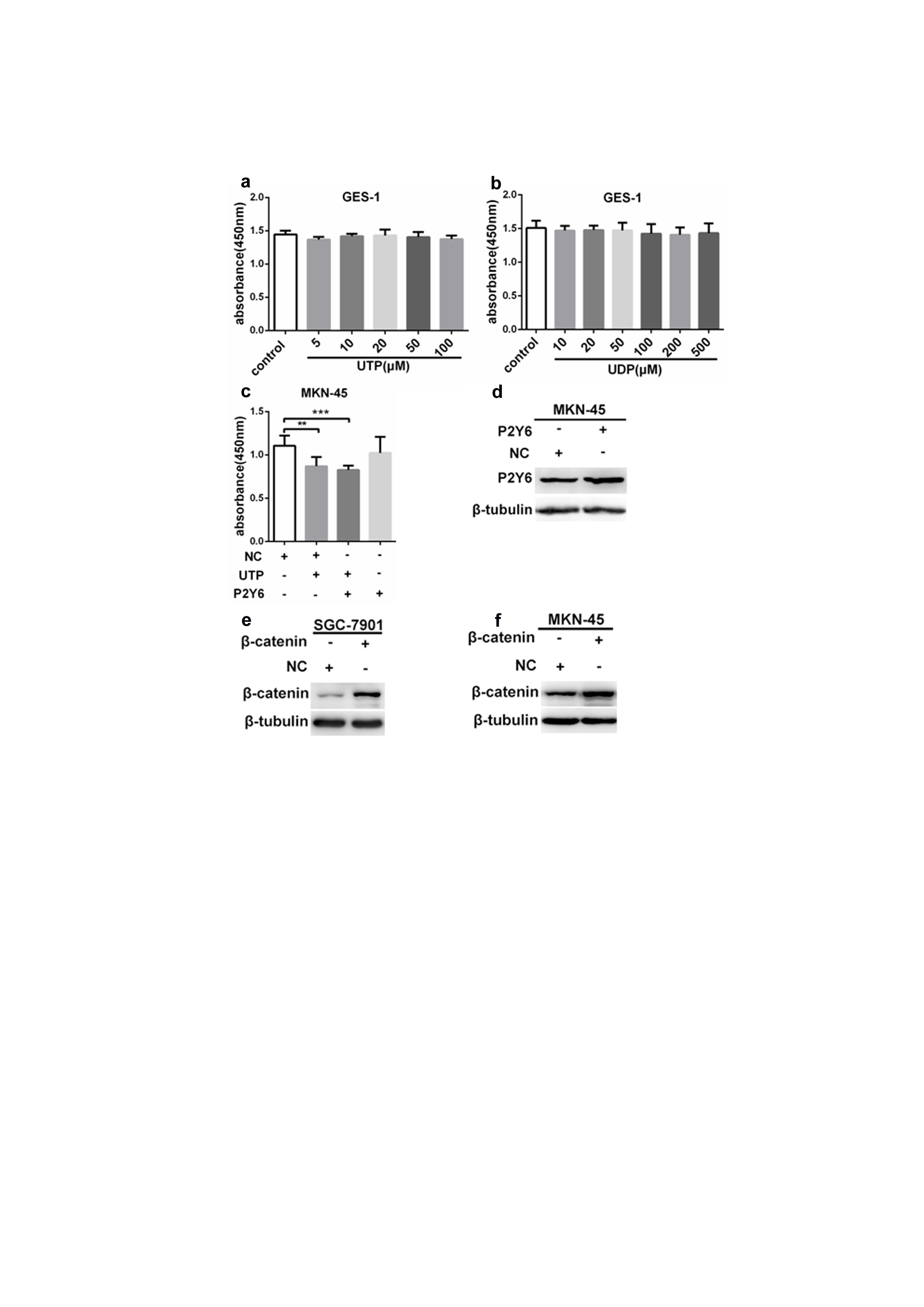


**Supplementary figure S4. Over-expression of P2Y6 and β-catenin in gastric cancer cells. a&b:** No effects of UTP (20 µM) and UDP (50 µM) on proliferation of GES-1 cells. **c:** UTP strongly inhibited proliferation of MKN-45 cells with over-expressed P2Y6 receptors. **d:** Western blot reveals the successful over-expression of P2Y6 receptors in MKN-45 cells. **e-f:** the successful over-expression of β-catenin in MKN-45 and SGC-7901 cells.


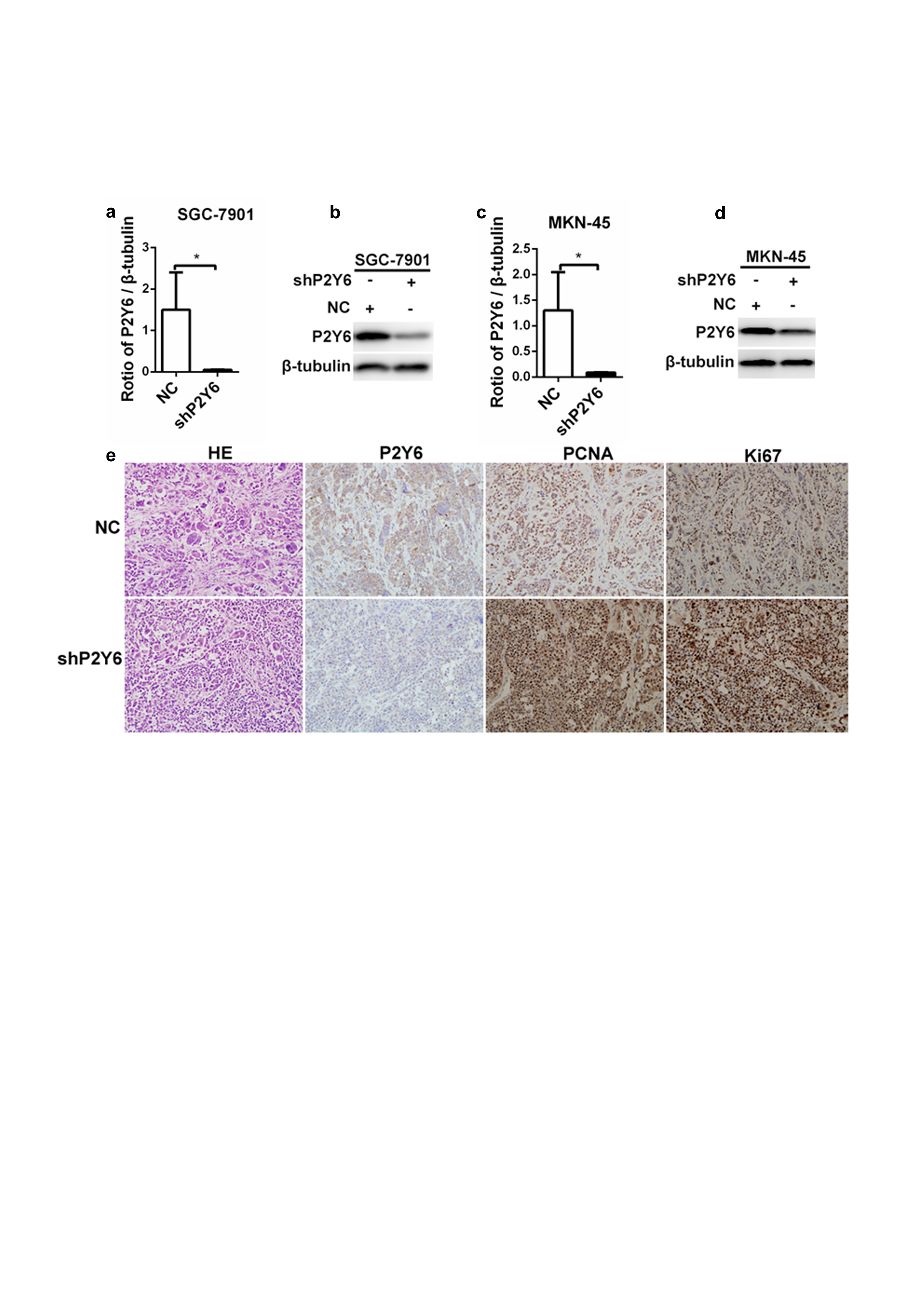


**Supplementary figure S5.** **Expression of P2Y6, PCNA and Ki-67 in gastric cancer cells. a-b:** RT-PCR and Western blot analysis reveals the successful knockdown of P2Y6 receptors in SGC-7901 cells. **c-d:** The successful knockdown of P2Y6 receptors in MKN-45 cells. **e:** Both HE and immunohistochemical staining analysis of P2Y6 receptors, PCNA and Ki-67 on the tumor sections obtained from nude mice. *P<0.05, n=3.
